# Functional *FOXC1* variants in familial and sporadic atrial septal defect with cellular and animal validation

**DOI:** 10.1002/ctm2.1676

**Published:** 2024-06-25

**Authors:** Huan‐Xin Chen, Hai‐Tao Hou, Xiu‐Li Wang, Jun Wang, Qin Yang, Guo‐Wei He

**Affiliations:** ^1^ Department of Cardiac Surgery and The Institute of Cardiovascular Diseases TEDA International Cardiovascular Hospital, Tianjin University and Chinese Academy of Medical Science Tianjin China; ^2^ Tianjin Key Laboratory of Molecular Regulation of Cardiovascular Diseases and Translational Medicine Tianjin China

Dear Editor,

Newborns are prone to morbidity and mortality due to heart and/or great vessel structural abnormalities, which is known as congenital heart disease (CHD).^1^ Atrial septal defect (ASD) is one of the most common CHDs and occurs in isolation or in combination with other complex cardiac malformations.^2^ The pathogenesis of ASD is heavily influenced by genetic defects,^3^ but the genetic determinants of ASD are unclear.

We have found a family including 30 members with four of them suffering from ASD who were operated on at our hospital (Figure 1A). We hypothesized that the family had common genetic variants. The present study was designed to discover the genetic variants causing familiar ASD and to reveal whether the discovered variants are also common in other sporadic ASD patients. Further, the function of the genetic variants was examined at the cellular level and CRISPR/Cas9 was used to construct variant‐specific mutant mice in order to observe the abnormality of the heart structure. These investigations were undertaken to identify the specific genetic variants causing ASD in order to provide insights into the aetiology of ASD.

**FIGURE 1:**
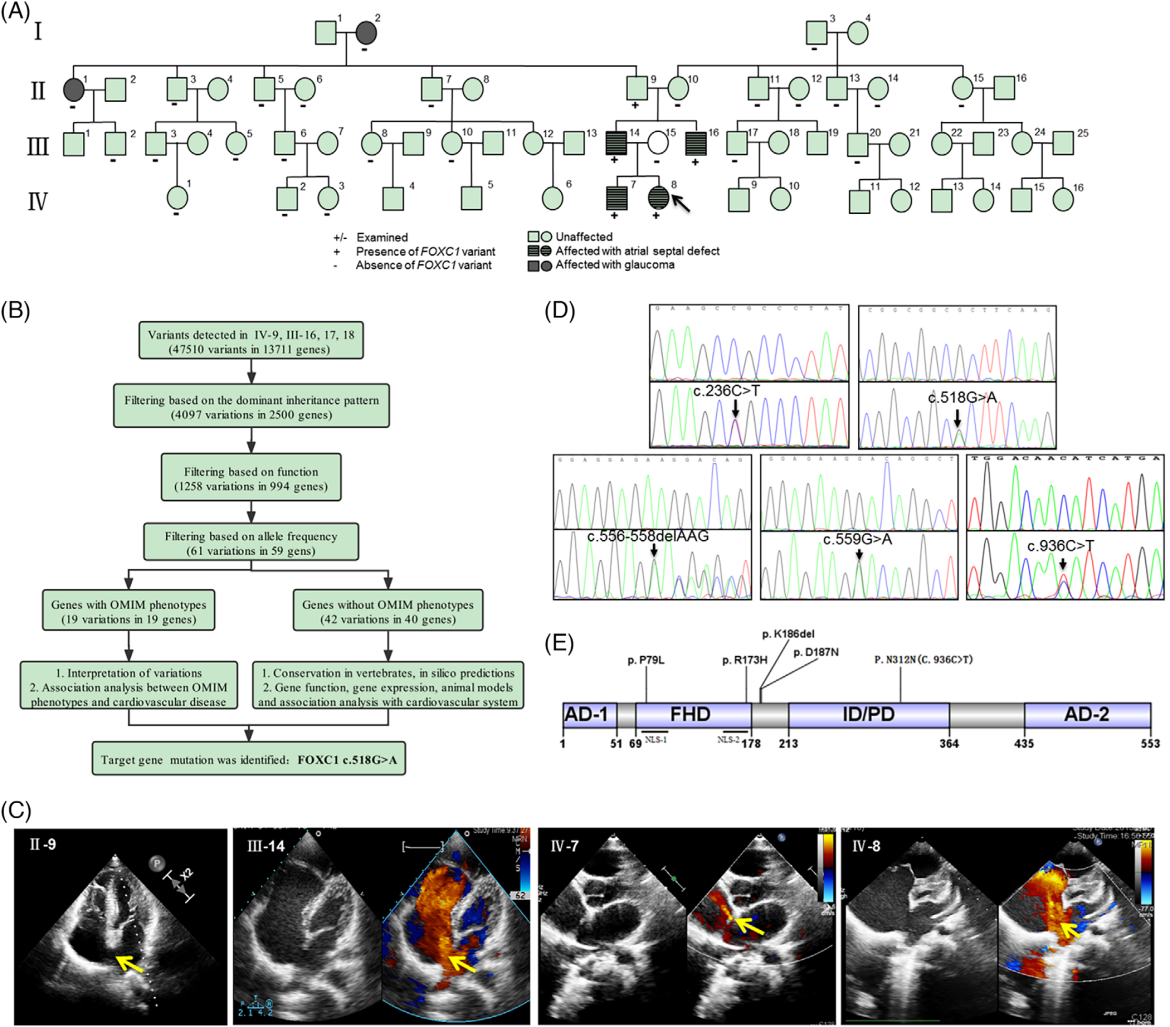
The information on family pedigree with atrial septal defect (ASD) and the identified *FOXC1* variant in the present study. (A) The inheritance of ASD in multiple members of a single family. Cases are numbered from left to right by generation. The proband (IV‐8) is indicated by an arrow. Squares represent male family members, and circles represent female family members. Affected family members are indicated by shadings. (B) The flowchart of variant identification by WES. (C) The echocardiographic diagnosis for four ASD patients: II‐9, III‐14, IV‐7 and IV‐8. The arrow indicates the atrial septal defect, except in II‐9, in whom the septum was extremely thin and was not seen almost forming a defect. (D) Chromatograms of the variants. Five heterozygous variants were validated by the Sanger sequence. The top panels show wild type, and the bottom panels show heterozygous variants. Variants are marked with arrows. (E) The functional domains of FOXC1 and the location of *FOXC1* variants. AD, transcriptional activation domain; FHD, forkhead domain; ID/PD, transcriptional inhibitory domain/phosphorylation domain; NLS, nuclear localization signals.

In Stage I, whole exome sequencing (mean read depth = 80×, GRCh37/hg19) was performed in 4 subjects from the family at BGI Clinical Laboratories (Figure 1B), including the proband (IV‐8), her affected father (III‐14) and uncle (III‐16), and her unaffected mother (III‐15) (Figure 1A). A heterozygous missense variant, *FOXC1* c.518G > A (p.R173H) was identified in three ASD patients (IV‐8, ostium secundum defect; III‐14, sinus venosus defect; Table S1). Subsequently, the *FOXC1* code region was sequenced with the Sanger sequencing in other family members revealing that the proband's grandfather (II‐9) and brother (IV‐7) had R173H. Doppler echocardiography indicated that IV‐7 had ostium secundum ASD and II‐9 had a very thin interatrial septum at the mid part of the septum (Figure 1C).

In Stage II, a new cohort of 335 unrelated sporadic and isolated ASD patients was enrolled to screen the *FOXC1* variants by Sanger sequencing. The primer pairs designed are shown in Table S2. As a result, other four heterozygous variants were identified in four isolated ASD patients, including three deletion/missense variants: c.556‐558delAAG (p.K186del), c.559G > A (p.D187N), c.236C > T (p.P79L) and a synonymous variant c.936C > T (p.N312 = ) (Figure 1D). All these 4 patients had ostium secundum ASD (Table S1).

*FOXC1* (NM_001453.3) belongs to the winged helix/forkhead transcription factor family, located at 6p25.^4^ The forkhead domain (FHD) is the core region of FOXC1 with a sequence of 110 amino acids and is evolutionarily conserved and exists in a wide range of species (Figure 1E).^5^ As a transcription factor, FOXC1 plays a key role in embryonic stem cells differentiating into functional cardiomyocytes and the occurrence of congenital heart disease, through proper regulation of specific downstream gene networks in various biological processes.^6^

The above four heterozygous deletion/missense variants (p.R173H, p.P79L, p.K186del and p.D187N) are mainly distributed in and near the FHD region in FOXC1 (Figure 1E). The allele frequency from gnomAD shows that these four variants are extremely rare in all populations recorded (Table S3). The predicted results by using bioinformatics tools (Polyphen‐2, Mutation taster and SIFT) showed that the amino acids positions 79, 173, 186 and 187 of FOXC1 were highly conserved and these variants were probably pathogenic, especially R173H and P79L (Tables S4 and S5).

In Stage III, the biological functions of the variants were elucidated in cell and mouse models.

The FOXC1 protein regulates the expression of *Tbx1*, which is necessary for mesoderm formation and plays a critical role in cardiac development.^7^ The coding region of *FOXC1* was cloned into the plasmid pCDNA3.1(+). Site‐directed mutagenesis was used to construct the mutants. The *TBX1* promoter was amplified and sub‐cloned into the pGL3‐basic reporter plasmid. The plasmids were co‐transfected into HeLa cells. In the R173H and P79L groups, the transcriptional activity of the *TBX1* promoter was significantly lower than the wild type (*p* < .05, t‐test) as demonstrated by the results of the dual‐luciferase reporter assay (Figure 2A).

**FIGURE 2:**
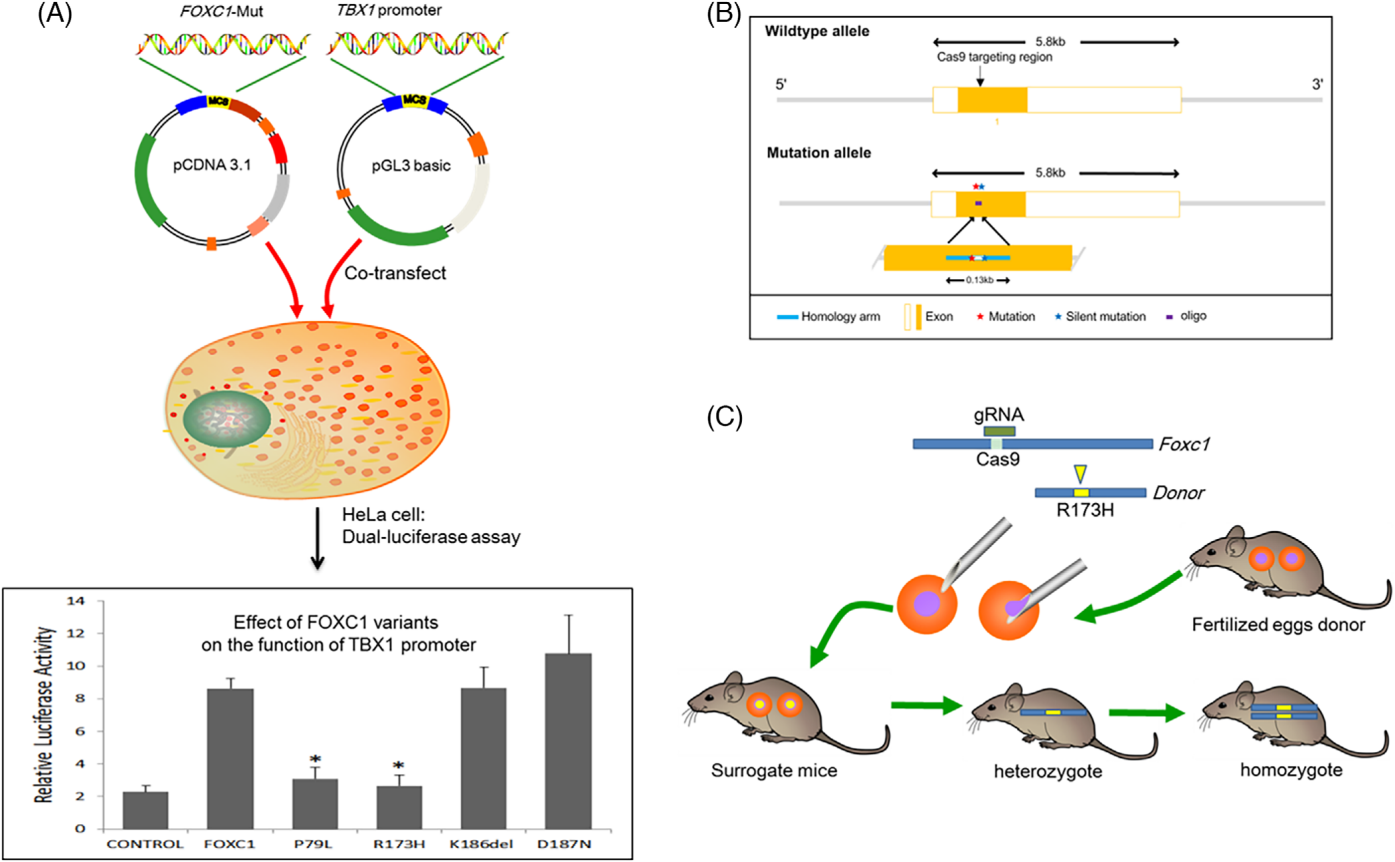
The change of transcriptional activity of FOXC1 variants and schematic diagram of transgenic mice for CRISPR/Cas9 in the present study. **(A)** Relative transcriptional activity of the *TBX1* promoter was regulated by *FOXC1* with or without variants in HeLa cells. Compared to the wild type, the transcriptional activity was down‐regulated by P79L and R173H, and up‐regulated by D187N; K186del did not alter the activity. *, *p* < .05. **(B, C)** The schematic diagram shows the construction of the site‐specific mutant mice.

The *FOXC1* R173H was “co‐occurrence” with the ASD phenotype in five family members in an autosomal dominant pattern.

As a mouse model, we constructed the *Foxc1* (NM_008592.2) R173H site‐specific mutant mice by CRISPR/Cas9‐mediated genome engineering to observe the structure of the atrial septum (Figure 2B,C, Figure 3A and Table S2).

**FIGURE 3:**
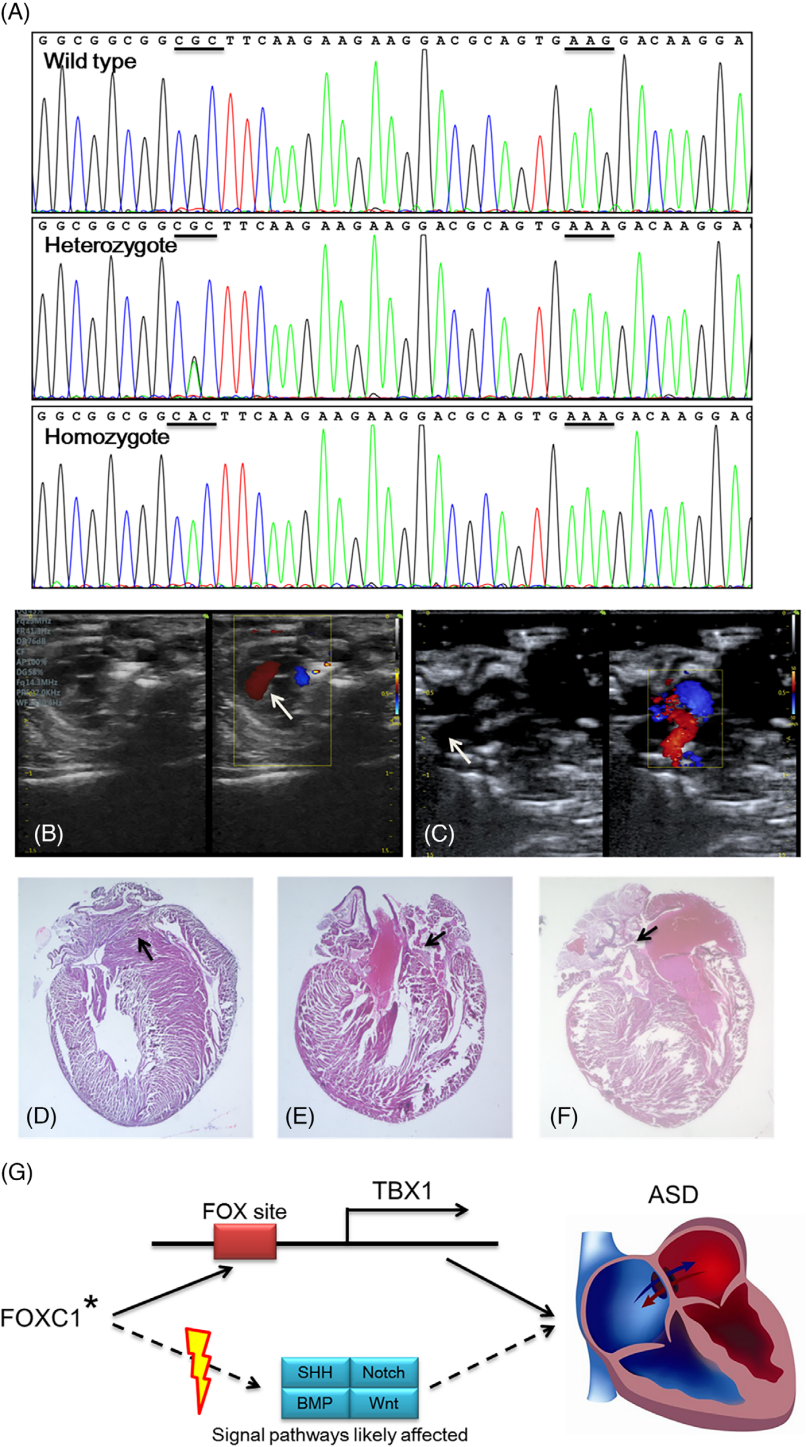
Variant‐specific mutant mice were constructed, then analyzed by Doppler and histological, and the role of FOXC1 involved in heart development. **(A)** Sanger sequencing was used to detect the genotype of mice. The point mutation (CGC–CAC) was introduced to form heterozygote or homozygote mice and the silent mutation (AAG–AAA) was designed to prevent the binding and re‐cutting of the sequence by gRNA after homology‐directed repair. **(B, C)** The echocardiography of wild‐type (*n* = 34) (**B**) and homozygous site‐specific mutant (**C**) mice (*n* = 18). The arrow shows the atrial septum. **(D, E)** The morphological structure of heart coronal sections of wild type (*n* = 4) and homozygous site‐specific mutant mice (*n* = 6), H&E stain, 10×. The arrow indicates the normal septum in wild‐type mice (**D**) and the thin atrial septum of the heart in mutant mice (**E, F**). **(G)** The schematic diagram of FOXC1 involved in heart development. FOXC1 directly regulates the downstream genes (*TBX1*) or as part of signalling pathways (SHH, BMP, Notch and Wnt) plays a key role in heart development. *, gene variant; SHH, Sonic Hedgehog; BMP, Bone morphogenetic protein; ASD, atrial septal defect.

Echocardiography (*Foxc1* ^R173H/+^, *n* = 40; *Foxc1* ^R173H/R173H^, *n* = 18) showed that the atrial septum in homozygous transgenic mice became thin compared to wild‐type mice (*n* = 34) (Figure 3B,C). Further, histological examination was performed in the paraffin tissue sections of 10 mice hearts (*Foxc1* ^R173H/R173H^, *n* = 6; wild type, *n* = 4) with H&E staining and two homozygous transgenic mice had significantly thinner atrial septum compared to the wild mice (Figure 3D,F). In total, the difference was statistically significant (three in 24 *Foxc1* ^R173H/R173H^ vs. 0 in 38 wild‐type mice, *p* = .025, Chi‐square). This significantly thinner atrial septum formed the pathological basis for the development of ASD. The above phenotype of three mice was similar to the family member II‐9. The phenotype of the thin atrial septum is more pronounced in homozygous mice than in heterozygous mice. In the latter (*n* = 40), the thin atrial septum was not found.

In summary, the present study identified five heterozygous variants in *FOXC1* from ASD patients in Chinese population, including c.236C > T (p.P79L), c.518G > A (p.R173H), c.556_558delAAG (p.K186del), c.559G > A (p.D187N) and c.936C > T (p.N312 =). To our knowledge, these five variants have not been reported previously in either familial or sporadic ASD. Functional analysis confirmed that the R173H and P79L variants led to significant changes in the FOXC1 transcriptional activity. In addition, the R173H affected the formation of atrial septum in site‐specific mutant mice. Thus, functional variants of *FOXC1* found in this study most likely cause cardiac anomalies in humans. The identification of variants further supports the important pathological role of the transcription factor FOXC1 in ASD. Figure 3G illustrates that FOXC1 directly regulates the downstream genes (*TBX1*) or as part of signalling pathways (SHH, BMP, Notch and Wnt) plays a key role in heart development, finally causing heart defects.^8^, ^9^, ^10^ This study provides a new insight into the genetic causes, diagnosis, and counselling of familial and sporadic ASD.

## AUTHOR CONTRIBUTIONS

Guo‐Wei He and Huan‐Xin Chen conceptualized the study; Huan‐Xin Chen conducted the experiments; Qin Yang contributed to the discussion of the protocol and analysis of the results; Hai‐Tao Hou, Xiu‐Li Wang and Jun Wang collected the blood samples from familial and sporadic atrial septal defect patients; Huan‐Xin Chen and Guo‐Wei He prepared the original draft of the manuscript; Guo‐Wei He had overall responsibility for the present study. All authors approved the final manuscript version.

## CONFLICT OF INTEREST STATEMENT

The authors declare no conflict of interest.

## FUNDING INFORMATION

This work was supported by the National Natural Science Foundation of China [82170353 and 82370350]; Tianjin Municipal Science and Technology Bureau [22ZYQYSY00020]; TEDA International Cardiovascular Hospital Internal Grant [2023‐TD‐001], and Tianjin Key Medical Discipline (Specialty) Construction Project (TJYXZDXK‐019A).

## ETHICS STATEMENT

The study was reviewed and approved by the Institutional Review Board of TEDA International Cardiovascular Hospital (2021‐0715‐4). An informed consent form was obtained from all subjects or their parents or guardians.

## Supporting information

Supporting Information

## Data Availability

The datasets used and/or analyzed during the current study are available from the corresponding author upon reasonable request.
