# Supplementary material for: Functional FOXC1 variants in familial and sporadic atrial septal defect with cellular and animal validation
Source: Clin Transl Med. 2024 Jun 25;14(7):e1676. doi: 10.1002/ctm2.1676 (PMC11199059; doi:10.1002/ctm2.1676)
Supplement: Supplementary file 1 — Supporting Information [file CTM2-14-e1676-s001.docx]

**Supplemental Table 1. The ASD type of each patient with variant.**

| Family members with variant c.518G>A (p.R173H) | Type of ASD |
| --- | --- |
| The proband (IV-8) | Ostium secundum |
| The proband's father (III-14) | Sinus Venosus |
| The proband's uncle (III-16) | No information |
| The proband's brother (IV-7) | Ostium secundum |
| The proband's grandfather (II-9) | Very thin atrial septum |
| The sporadic and isolated ASD patients with variants | Type of ASD |
| c.556-558delAAG (p.K186del) | Ostium secundum |
| c.559G>A (p.D187N) | Ostium secundum |
| c.236C>T (p.P79L) | Ostium secundum |
| c.936C>T (p.N312=) | Ostium secundum |

**Supplemental Table 2. Sequence of primers and nucleotides designed in the study.**

|  | | Primer | | Location |
| --- | --- | --- | --- | --- |
| *FOXC1* in Human | | | | |
| PCR | P-F1: 5’-ACGGATGCTCAAAAGTTCAGAAG-3’  P-R1: 5’-TTCTCGGTCTTGATGTCCTGGAT-3’  P-F2: 5’-CATCTACCAGTTCATCATGGACCG-3’  P-R2: 5’-AAGTGGAGGTGGCTCTGAATTAAT-3’ | | | 4463-4485  5666-5688  5308-5331  6861-6884 |
| Sequencing | S-F1: 5’-CATCTACCAGTTCATCATGGACCG-3’  S-F2: 5’-CGCATCCAGGACATCAAGACC-3’  S-F3: 5’-GACTACTCTCTGCCTCCGGTC-3’  S-R1: 5’-CGGTCCATGATGAACTGGTAGAT-3’  S-R2: 5’-TTCTCGGTCTTGATGTCCTGGAT-3’ | | | 5308-5331  5663-5683  6287-6307  5309-5331  5666-5688 |
| *Foxc1* in site-specific mutant mice | | | | |
| Single-guide RNA | | | CAAGAAGAAGGACGCAGTGAAGG | |
| Donor oligo | | | ACGCTCGACCCGGACTCCTACAACATGTTCGAGAACGGCAGCTTCCTGCGGCGGCGGCGGCACTTCAAGAAGAAGGACGCAGTGAAAGACAAGGAGGAGAAGGGCCGGCTGCACCTCCAAGAACCGCCACCGC | |
| Genotype | | | TF: 5’-CTTCTATCGGGACAATAAGCAGGGCT-3’  TR: 5’-TGCTCCCGCTCGACAAGCTG-3’ | |

**Supplemental Table 3. Allele Freguency of 4 variants in gnomAD (V3.1.2).**

| Population | Allele Freguency in gnomAD | | | | | | |
| --- | --- | --- | --- | --- | --- | --- | --- |
|  | c.518G>A (p.R173H) | c.556-558delAAG (p.K186del) | c.559G>A (p.D187N) | c.236C>T (p.P79L) | | c.936C>T (p.N312=) | |
| East Asian | None | None | 0.000 | | None | | 0.001746 |
| European (non-Finnish) |  |  | 0.000 | |  |  | 0.00001483 |
| African/African American |  |  | 0.000 | |  |  | 0.000 |
| Latino/Admixed American |  |  | 0.000 | |  |  | 0.000 |
| Ashkenazi Jewish |  |  | 0.000 | |  |  | 0.000 |
| European (Finnish) |  |  | 0.000 | |  |  | 0.000 |
| Other |  |  | 0.000 | |  |  | 0.000 |
| South Asian |  |  | 0.001659 | |  |  | 0.000 |
| XX | None | None | 0.00001289 | | None | | 0.00005189 |
| XY |  |  | 0.00009454 | |  |  | 0.00008166 |
| Total | None | None | 0.00005276 | | None | | 0.00006642 |

**Supplemental Table 4. Prediction of variants in Polyphen-2, Mutation taster, SIFT.**

| Variant | Polyphen-2 | | Mutation taster | | SIFT | |
| --- | --- | --- | --- | --- | --- | --- |
|  | Score | Predicted result | Score | Predicted result | Score | Predicted result |
| p.P79L | 1.000 | probably damaging | 0.9999 | disease causing | 0.00 | Affect protein function |
| p.R173H | 0.998 | probably damaging | 0.9999 | disease causing | 0.00 | Affect protein function |
| p.K186del | -- | -- | 0.9992 | disease causing | -- | -- |
| p.D187N | 0.029 | Benign | 0.7416 | disease causing | 0.00 | Affect protein function |

**Supplemental Table 5. Conservation of variants at amino acid positions 79, 173, 186 and 187 in coding sequence of FOXC1.**

| **Species** | **Gene** | **p.P79L** | **p.R173H** | **p.K186del/p.D187N** |
| --- | --- | --- | --- | --- |
| Human | ENST00000380874 | MVKPPYS | RRRRFKK | KEEKDRLH |
| M. mulatta | ENSMMUG00000005778 | MVKPPYS | RRRRFKK | KEEKDRLH |
| F. catus | ENSFCAG000000502 | MVKPPY | RRRRFKK | KEEKDRLH |
| M. musculus | ENSMUSG00000050295 | MVKPPYS | RRRRFKK | KEEKGRLH |
| T. rubripes | ENSTRUG00000011513 | MVKPPYS | RRRRFKK | KEERLQKD |
| D. rerio | ENSDARG00000091481 | MVKPPYS | RRRRFKK | KEDRG- - - |
| D. melanogaster | FBgn0014143 | IVKPPYS | RRRRFKK | KEE - - - - - |
| X. tropicalis | ENSXETG00000000594 | MVKPPYS | RRRRFKK | KEDKDRL - |
